# Investigation of liposomal self-adjuvanting peptide epitopes derived from conserved blood-stage Plasmodium antigens

**DOI:** 10.1371/journal.pone.0264961

**Published:** 2022-03-11

**Authors:** Md. Tanjir Islam, Mei-Fong Ho, Ummey J. Nahar, Ahmed O. Shalash, Prashamsa Koirala, Waleed M. Hussein, Danielle I. Stanisic, Michael F. Good, Mariusz Skwarczynski, Istvan Toth

**Affiliations:** 1 School of Chemistry and Molecular Biosciences, The University of Queensland, St Lucia, QLD, Australia; 2 Institute for Glycomics, Griffith University, Southport, QLD, Australia; 3 School of Pharmacy, The University of Queensland, Woolloongabba, QLD, Australia; 4 Institute for Molecular Biosciences, The University of Queensland, St Lucia, QLD, Australia; Universidade Federal de Minas Gerais, BRAZIL

## Abstract

Malaria is a vector born parasitic disease causing millions of deaths every year. Despite the high mortality rate, an effective vaccine against this mosquito-borne infectious disease is yet to be developed. Up to date, RTS,S/AS01 is the only vaccine available for malaria prevention; however, its efficacy is low. Among a variety of malaria antigens, merozoite surface protein-1(MSP-1) and ring-infected erythrocyte surface antigen (RESA) have been proposed as promising candidates for malaria vaccine development. We developed peptide-based *Plasmodium falciparum* vaccine candidates that incorporated three previously reported conserved epitopes from MSP-1 and RESA into highly effective liposomal polyleucine delivery system. Indeed, MSP-1 and RESA-derived epitopes conjugated to polyleucine and formulated into liposomes induced higher epitope specific antibody titres. However, immunized mice failed to demonstrate protection in a rodent malaria challenge study with *Plasmodium yoelii*. In addition, we found that the three reported *P*. *falciparum* epitopes did not to share conformational properties and high sequence similarity with *P*. *yoelii* MSP-1 and RESA proteins, despite the epitopes were reported to protect mice against *P*. *yoelii* challenge.

## Introduction

Malaria is the 5th leading cause of death worldwide among infectious diseases [1]. Around 229 million people were infected by the disease globally in 2019, with 409,000 of those infections resulting in death [2]. Recently, World Health Organization (WHO) has approved the first malaria vaccine, RTS,S/AS01, despite its relatively poor protective efficacy (30–40%) [3]. Peptide-based subunit vaccines have been investigated for malaria, as they are easier to produce than traditional whole parasite-based vaccines [4], however peptide antigens suffer from poor immunogenicity. To overcome this problem, multiple epitopes (B and T-cell) can be incorporated in linear peptides to enhance the immunogenicity [5], along with an adjuvant and/or delivery system [6].

**Fig 1:**
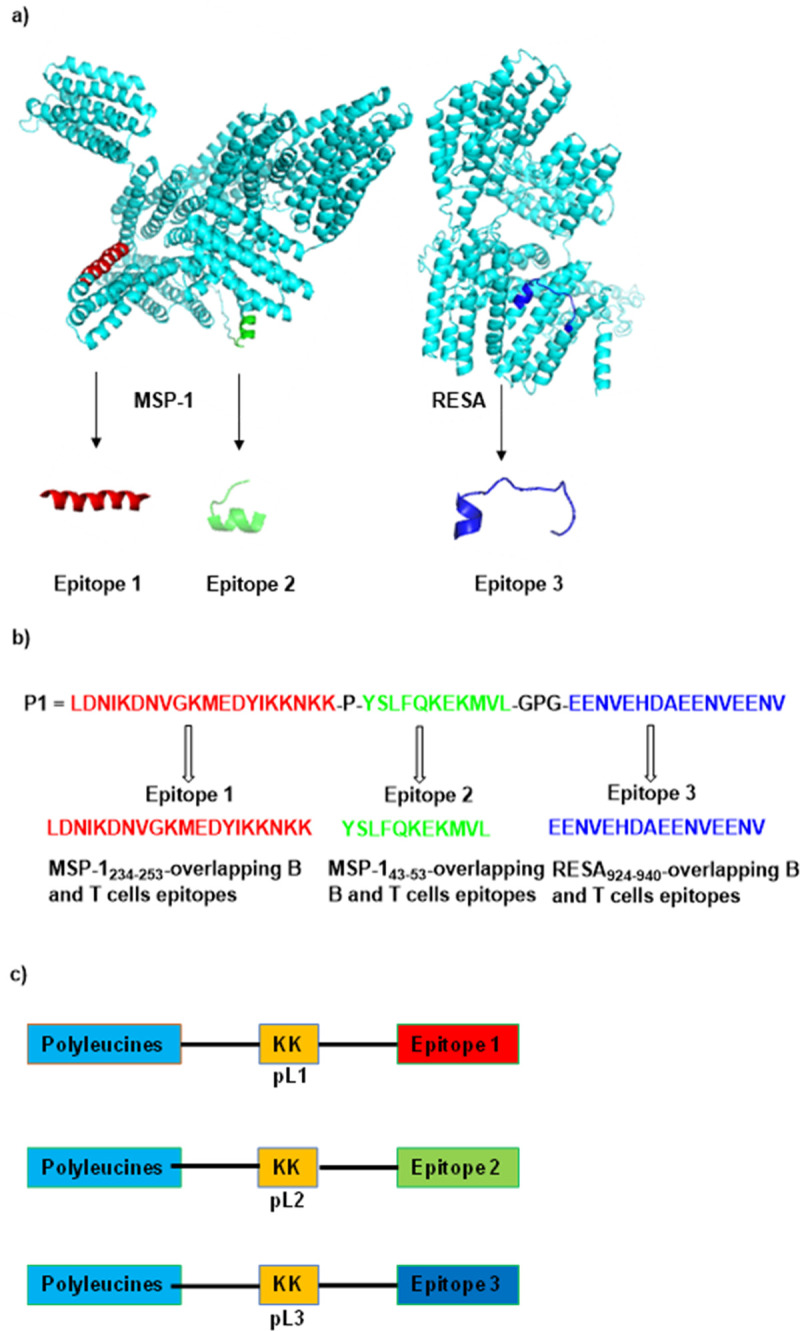
Malaria antigens, B and T-cell epitopes and polyleucine conjugated epitopes. a) three-dimensional structure of MSP-1 and RESA according to molecular modelling (models were generated from I-TASSER online server, https://zhanglab.dcmb.med.umich.edu/I-TASSER/); b) epitopes 1–3 and multiple epitope peptide **P1**; c) **pL1**, **pL2**, and **pL3** vaccine constructs composed of epitopes 1, 2 and 3, respectively; bearing a 10-unit polyleucine tail and two lysine amino acids as a spacer.

Merozoite surface protein-1 (MSP-1) and ring-infected erythrocyte surface antigen (RESA) are the two potent antigens expressed by the blood-stages of human malaria parasite, *Plasmodium falciparum*. Several epitopes (both B and T-cells) from MSP-1 and RESA were found to be conserved in *P*. *falciparum* [7,8]. A multiple antigen peptide (**P1**, Fig 1A and 1B) derived from *P*. *falciparum’s* MSP-1 and RESA was immunogenic in mice when administered with adjuvants [9,10]. Furthermore, sera from *P*. *yoelii* infected mice recognized or cross-reacted with **P1** when examined by enzyme-linked immunosorbent assay (ELISA). Despite the fact that, **P1** peptide was derived from *P*. *falciparum* proteins, P1-immunized mice were protected when challenged with *P*. *yoelii* [10]. Five among 10 mice immunized with **P1**+CFA survived the malaria challenge. Surprisingly, in **P1**+alum mice group, eight out of ten mice survived the challenge [10], despite Alum is generally poorly effective when combined with peptides [11].

Peptide **P1** was composed of overlapping B and T-cell epitope 1 (LDNIKDNVGKMEDYIKKNKK), B-cell epitope 2 (YSLFQKEKMVL) and overlapping B and T-cell epitope 3 (EENVEHDAEENVEENV) (Fig 1B). Epitopes 1 and 2 were derived from the N-terminal conserved region of MSP-1 and epitope 3 was derived from the N-terminal conserved region of RESA (Fig 1A).

A variety of delivery systems have been investigated to improve the immunogenicity of peptide vaccines. The purpose of these delivery systems is to avoid vaccine antigen loss due to degradation *in vivo* and to effectively deliver vaccine constructs to immune cells [12]. Polyhydrophobic amino acids (e.g. polyleucine) conjugated to peptide epitopes (B and T-cell) demonstrated excellent capacity to enhance immunogenicity [13]. Upon coupling with peptide antigens, poly(hydrophobic amino acids) self-assembled into nanoparticles and were able to stimulate the production of high antibody titres in mice. Polyleucine conjugated vaccine constructs were found to be effective against *Streptococcus pyogenes* bacteria and hookworm parasites infection in challenge experiments [13–15]. Liposomal formulations are often used as biodegradable, non-toxic carriers with the ability to stimulate cellular and humoral immunity in mice and humans [16–18]. In addition, mannosylated liposomes have been used as a highly effective delivery system in the development of whole parasite blood-stage malaria and babesiosis vaccines [19–21].

Here, we aimed to develop a liposome-based vaccine carrying the previously reported epitopes 1–3 derived from *P*. *falciparum*, with improved efficacy and without the use of a toxic adjuvant (e.g., CFA). Epitopes 1, 2 and 3 from **P1** were conjugated to polyleucine (Fig 1C), and incorporated into the liposomal delivery system along with mannosylated ligands. The new vaccine candidates were tested for their ability to induce antibody production in immunized mice and to protect the mice against parasite challenge (*P*. *yoelii*). The previously reported CFA-adjuvanted **P1**-based vaccine derived from *P*. *falciparum* protein MSP-1 and RESA were used as a control to compare our proposed vaccine constructs [10].

## Materials and methods

### Materials

All chemical materials used in this study were analytical grade unless stated otherwise. 1,2-Dipalmitoyl-sn-glycero-3-phosphoethanolamine (DPPC), cholesterol, tween-20, dimethyldioctadecylammonium bromide (DDAB), complete freund’s adjuvant (CFA), incomplete freund’s adjuvant (IFA), and o-phenylenediamine dihydrochloride (OPD) tablets were purchased from Sigma-Aldrich. p-methylbenzhydrylamine (MBHA)^.^ HCl resin was purchased from Novabiochem. *N*, *N’*-dimethylformamide (DMF), trifluoroacetic (TFA) acid, acetonitrile, protected (*tert*-butyloxycarbonyl) Boc-amino acids and 1-[bis(dimethylamino)methylene]-1*H*-1,2,3-triazolo[4,5-b]pyridinium3-oxid hexafluorophosphate (HATU) were purchased from Mimotopes. Chloroform, methanol, skim milk powder, and other solvents were purchased obtained from Merck. Goat antimouse IgG (H+L)-HRP conjugate secondary antibody was purchased from Bio-Rad, Australia).

Analytical reverse-phase high performance liquid chromatography (RP-HPLC) and preparative RP-HPLC were performed on a Shimadzu instrument using Vydac analytical (C4 and C18) and preparative (C4 and C18) columns. HPLC solvent A: 0.1% TFA in water and solvent B: 0.1% TFA in acetonitrile: water (90:10); flow rate: 1 mL/min for analytical RP-HPLC and 20 mL/min for preparative RP-HPLC; detection: UV absorbance at 214 nm.

## Methods

### Synthesis of malaria vaccine components

The malaria vaccine constructs (**P1**, **pL1**, **pL2**, and **pL3**) were synthesised on 4-methylbenzylamine^.^HCl resin (100–200 mesh; substitution degree: 0.59 mmol/g) by manual *tert*-butoxycarbonyl-solid phase peptide synthesis (Boc-SPPS) following the standard protocol [22,23]. All peptides were synthesised on a 0.2 mmol scale. Boc-protected L-amino acids (0.84 mmol, 4.2 equiv.) were activated with HATU (0.80 mmol, 4.0 equiv.) and DIPEA (1.04 mmol, 5.2 equiv.) for 5 min before being added to the resin. Coupling of amino acids was conducted for 30 min (1^st^ coupling) and 1 h (2^nd^ coupling), separately. The Boc-protecting group was removed by treatment with TFA for 2 x 1 min. The Boc-resin was washed with DMF between deprotection and coupling. Acetylation was performed after the first amino acid in a sequence was coupled to block the untreated groups on the resin, then again after the final amino acid to acetylate the N-terminus. The acetylation solution was composed of 5% DIPEA, 5% acetic anhydride and 90% DMF. Cleavage of the final peptide sequence from Boc resin was performed using HF (Hydrogen Fluoride) cleavage (10 mL/g of resin) and p-cresol (0.5 mL/g of resin) at -5 to 0°C for 2.5 h. After cleavage, the peptide was precipitated in diethyl ether and dissolved in 50:50 solvent, which was prepared using 50% of HPLC solvent A: (0.1% TFA/90.9% H_2_O) and 50% of HPLC solvent B (90% acetonitrile/0.1% TFA/9.9% H_2_O), then filtered through a polyethylene frit. The resulting solution was freeze-dried and purified by preparative HPLC (C4 column). Purification for preparative HPLC: 25–45% solvent B gradient over 50 min. Solvent A, H_2_O + 0.1% TFA; solvent B, 90% acetonitrile + 10% H_2_O and 0.1% TFA. The purified peptide was analysed with analytical HPLC (C4 column) and electrospray ionisation mass spectrometry (ESI-MS). Analytical HPLC was conducted at a flow rate of 1 mL/min with ultraviolet (UV) detection at a wavelength of 214 nm on a Vydac analytical HPLC C4 column. The molecular weight of the peptide was calculated by ChemDraw software20.0. The molecular weight of the final purified compound was checked by ESI-MS (S1–S5 Figs). Mannosylated lipid core linker (**ML-1**) and 1,2-dioleoyl-sn-glycero-3-phosphoethanolamine-*N*-[carboxy(polyethylene glycol)]-mannose (DOPE-PEG-mannose, **ML-2**) were synthesised according to previously reported methods [24–26].

**pL1**: Yield: 48%; MW: 3749.0; ESI-MS: [M+3H]^3+^ m/z 1250.5 (calcd. 1250.6), [M+4H]^4+^ m/z 938.3 (calcd. 938.2); HPLC t_R_ = 22.9 min (C4 column); purity ≥99% (S1 Fig).

**pL2**: Yield: 44%; MW: 2814; ESI-MS: [M+2H]^2+^ m/z 1408.6 (calcd. 1408.0), [M+3H]^3+^ m/z 939.2 (calcd. 939.0), [M+4H]^4+^ 705.1 (calcd. 704.5); HPLC t_R_ = 22.8 min (C4 column); purity = 98% (S2 Fig).

**pL3**: Yield: 40%; MW: 3314.0; ESI-MS: [M+2H]^2+^ m/z 1658.1 (calcd. 1658.0), [M+3H]^3+^ m/z 1104.7 (calcd. 1105.6), [M+4H]^4+^ m/z 829.4 (calcd. 829.5); HPLC: t_R_ = 28.5 min (C4 column); purity = 98% (S3 Fig).

**P1**: Yield: 51%, MW: 5902.0; ESI-MS: [M+4H]^4+^ m/z 1476.7 (calcd. 1476.5), [M+5H]^5+^ m/z 1181.4 (calcd. 1181.4); HPLC: t_R_ = 18.3 min (C4 column); purity = 98% (S4 Fig).

**ML-2:** Mannose-azide (8.3 mg, 25 μmol), DOPE-PEG-alkyne (5 mg, 1.2 μmol, 1 equiv.) and 42 mg of copper wire were added to 1 mL of DMF. The air in the reaction mixture was removed by nitrogen bubbling for 30 sec. The reaction was conducted in the dark and stirred for 3 h at 50°C under nitrogen gas. The copper wires were removed, and the reaction mixture was slowly added to water (4 mL) at a rate of 0.005 mL/min to form micelles by self-assembly. The solution was dialysed in a dialysis bag (2 kDa) overnight. Pure **ML-2** was obtained following lyophilisation. The molecular weight (4500) of pure **ML-2** was calculated by ChemDraw, which was detected by MALDI-TOF mass spectrometry (S5 Fig).

### Particle size analysis

The particle size distribution of the malaria vaccine components was measured using a laser particle analyser (Zeta sizer 2000E, Malvern Instruments Ltd., Malvern, UK). The emulsion was diluted 10 times, then transferred to disposable zeta sizer cuvettes before measurement at 25°C with a 173° light scattering angle [27].

### Circular dichroism (CD) and *Plasmodium* protein sequence analysis

Peptide secondary structures were analysed according to a previously reported procedure [28]. Peptide conformation was assessed by a Jasco J-710 circular dichroism spectropolarimeter (Jasco Corp., Japan). Polyleucine-conjugated peptides 1–3 were dissolved in phosphate-buffered saline (PBS) at a concentration of 0.1 mg/mL (pH 6). The solution was transferred into a specific 1 mm quartz cuvette for detection. The study was carried out in the range of λ 190–260 nm under nitrogen atmosphere. The spectra for the peptides were 5 nm bandwidth with a 50 nm/min scan rate. Peptide absorbance measures were carried out in triplicate and the data was averaged and plotted. Spectra were normalised and reported as the mean residual ellipticity (mdeg^.^cm^2.^dmol^-1^) versus wavelength (λ). Quantitative analysis of the secondary structure of the polyleucine compounds was measured by analysing the composition spectrum of a given compound to yield a ratio of each pure individual compound spectrum (such as pure α-helix, β-sheet, and random coil contribution) that was obtained from the polyleucine spectra according to the previously reported method [29].

MSP-1 and RESA protein sequences alignment between *P*. *falciparum* and *P*. *yoelii* were performed using ClustalW online server v2.1 (S7 and S8 Figs)

### Animal study and malaria parasites

This study was performed according to the Australian Code of Practice for the Care and Use of Animals for Scientific Purposes, 8th edition, 2013. All animal procedures and protocols were approved by the Griffith University Animal Ethics Committee (AEC): AEC Approval Number: GLY/07/20/AEC. Female BALB/c mice (6-8-weeks-old) were purchased from the Animal Resource Centre, Willeton, Western Australia. Animals were housed in a pathogen-free environment in the Institute for Glycomics animal facility at Griffith University for 2 weeks prior to immunization. *P*. *yoelii* 17X was initially obtained from Richard Carter (Edinburgh, UK) and was maintained by serial passaging through inbred and outbred mice.

### Formulation of vaccine candidates in liposomes

Liposomes were formulated according to the method reported previously [20]. Briefly, in a 5 mL round bottom flask, a mixture of 5 mg of DPPC, 2 mg of DDAB, 1 mg of cholesterol, and 1 mg equiv. of the desired vaccine construct were dissolved in chloroform/methanol mixture at a ratio of 9:1. The solvent was slowly evaporated under reduced pressure and a thin lipid layer was formed at the base of the flask. The flask was then connected to a vacuum overnight to remove excess solvent. The liposomes were hydrated for 20 min with 1 mL sterile warm phosphate buffer solution (PBS), and vortexed at regular intervals. Independent liposome batches were prepared to include the different vaccine constructs.

### Immunization of mice with the vaccine constructs

BALB/c female mice (7 mice/group) were used for the experiment. Each mouse was subcutaneously immunized with formulations containing 50 μg of peptide on day 0. Animals were given booster immunizations on days 21 and 42. **P1** was emulsified in CFA at a 1:1 ratio for the first immunization and boosted with same inoculum emulsified in IFA. Blood samples were taken from tail tip of each mouse the day before immunization and two weeks after each boost.

### Immunoassays and antibody titres

B cell-specific IgG antibody responses to the administered vaccine constructs in immunized mouse sera were analysed by enzyme-linked immunosorbent assay (ELISA), as reported previously [30,31]. Specifically, 96-well microtitre plates were coated with malaria peptide antigen (**P1**) and polyleucine-conjugated peptide antigen (**pL1**, **pL2** and **pL3**), separately, with carbonate coating buffer (pH = 9.6; 5 μg of peptide per plate). To block the uncoated active sites in the well, 0.5% skim milk/PBS-tween-20 buffer solution (pH 7.2) was used. All sera were diluted (1:1000) in PBS containing 0.05% skim milk/PBS-tween-20 buffer and incubated for 90 min at 37°C. Horseradish peroxidase-conjugated goat anti-mouse IgG/goat anti-human IgG (Sigma) was used as a secondary antibody in the ELISA. o-phenylenediamine dihydrochloride (OPD) was used as substrate for the enzymatic reaction. After washing the plates, OPD solution (100 μL/well) was added to the wells for 20 min. The absorbance was then measured by plate reader at 450 nm. IgG titres were identified as the lowest dilution in the wells with an optical density three times greater than the standard deviation compared to the average value of the optical density of the sera collected from pre-immunized mice.

### Challenge study

The malaria challenge study was conducted in mice immunized with *P*. *falciparum*-derived epitopes followed by *P*. *yoelii* infection according to the previously reported method [32]. Mice were bled from tail tip and sera collected 14 days after the second vaccine boost. On the same day, the mice were intravenously infected with an inoculum of 1×10^5^ *Plasmodium yoelii* 17X infected erythrocytes (200 μL/mouse). The mice were monitored post-challenge. Daily clinical scores of 0 to 4 were assigned based on pallor, posture, activity and activity to external stimuli, fur texture and urine colour. Mice that showed signs of severe distress, according to criteria on the clinical scoresheet, or those that experienced >15% weight loss from the time of challenge, were euthanized using CO_2_ gas or by cervical dislocation.

The blood was collected every 2 days following challenge and the average weight and hemoglobin were measured every 4 days. Blood smears were collected from the tail tip of each mouse. Slides were air-dried, fixed in methanol and stained with Giemsa stain. Peripheral blood parasitemias were determined by counting blood smears. A Hemocue Hb 201+ Analyser was used to measure haemoglobin and a weight balance used to measure weight of each mouse separately.

## Results

### Synthesis and characterisation of the vaccine candidates

**Fig 2:**
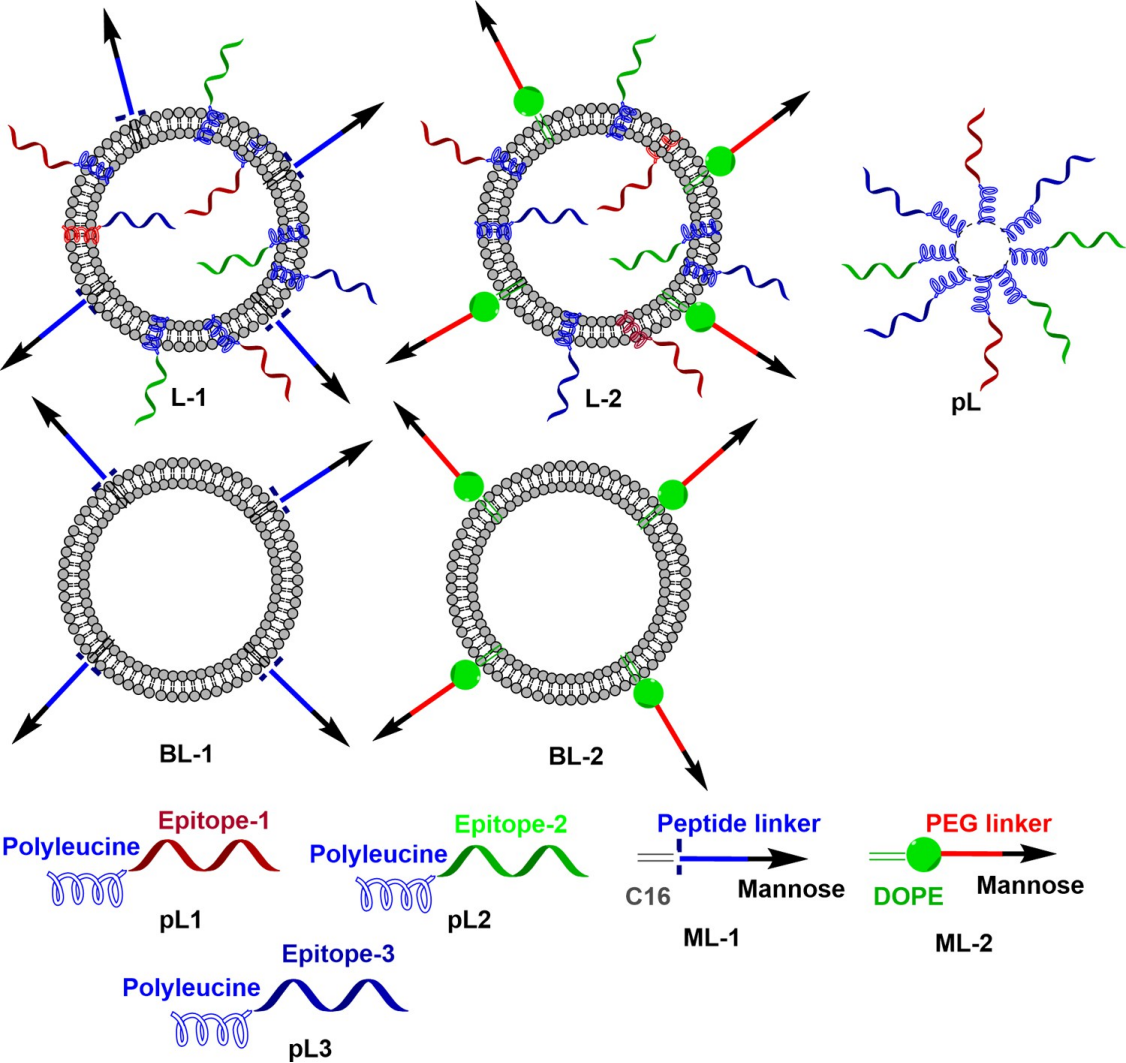
Schematic representation of the liposomal vaccine candidates. **L-1** and **L-2**, bearing polyleucine conjugated epitopes (**pL1-3**) and **ML-1** and **ML-2** targeting moieties, respectively; the mixture of polyleucine-conjugated epitopes, **pL**; and the blank liposomes, **BL-1** and **BL-2**.

The new vaccine candidates were designed by conjugating polyleucine attached to the peptide epitopes 1, 2 and 3 separately and anchored into mannosylated liposomes (Figs 1C and 2). Vaccine constructs **pL1**, **pL2** and **pL3** conjugates were first synthesised by Boc-SPPS. The mannose receptor targeting moiety, **ML-1**, was produced as a conjugate of mannose, a short peptide linker (SSKK) and lipid (C16; 2-aminohexadecanoic acid) [20,21], while **ML-2** was produced as a conjugate of mannose, PEG linker, and DOPE lipid [26]. Cationic liposomes were produced by lipid film hydration using DPPC, DDAB and cholesterol, as described previously [33]. All the polyleucine conjugated epitopes (**pL1-pL3**) and mannose conjugates (**ML-1** and **ML-2**) were incorporated during the thin film formation step. As each liposomal formulation contained **pL1**, **pL2** and **pL3**, a mixture of the three polyleucine-conjugated epitopes (**pL**) was self-assembled to serve as a control (Fig 2). Blank liposomes, **BL-1**, and **BL-2** anchored only with mannosylated lipids (**ML-1** and **ML-2**) were also formulated as control (Fig 2).

The particle size and polydispersity index (PDI) of **L-1**, **L-2**, **BL-1**, and **BL-2** were analysed by dynamic light scattering (Table 1, S6 Fig). The size of the blank liposomes, **BL-1** and **BL-2** was around 140 nm and the PDI was below 0.1. After anchoring **pL1-pL3** and **ML-1** or **ML-2** into the liposomes, size and PDI increased slightly, as expected. **pL** generated larger nanoparticles with very broad size distribution.

**Table 1:** Average particle size and PDI of vaccine constructs analysed by dynamic light scattering.

Liposomes	Diameter (nm) ± STD	PDI ± STD
**BL-1**	142 ± 1	0.06 ± 0.01
**BL-2**	141 ± 2	0.08 ± 0.02
**L-1**	175 ± 2	0.24 ± 0.04
**L-2**	162 ± 2	0.18 ± 0.03
**pL**	293 ± 13	1.00 ± 0.30

**Fig 3:**
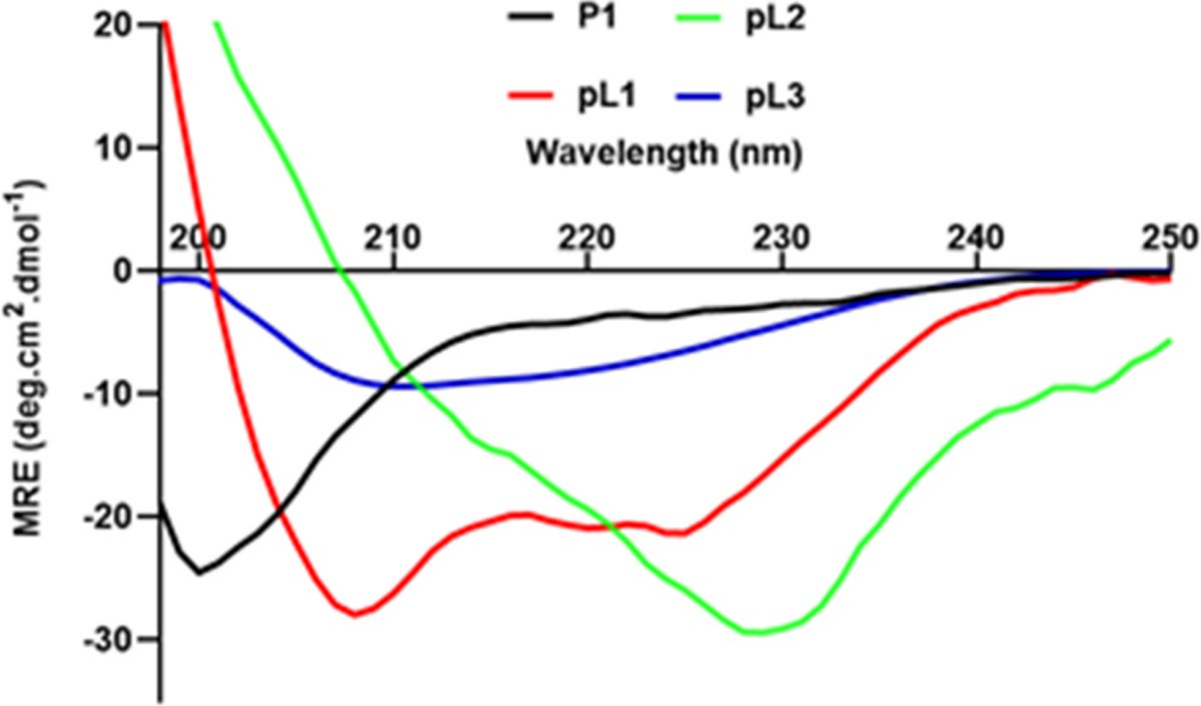
Normalised circular dichroism (CD) spectra of peptides P1, pL1, pL2 and pL3.

The conformation of **P1**, **pL1**, **pL2** and **pL3** was analysed by circular dichroism (CD) spectroscopy (Fig 3). A more random (78%) than α-helical (22%) conformation was observed for **P1** peptides. Polyleucine-conjugated peptide **pL1** formed as 72% α-helical and 27% random coil. Peptide **pL2** adopted mainly a β-sheet conformation (72% β-sheet, 26% α-helical and 1% random coil). The third peptide **pL3** adopted both β-sheet and α-helical conformations (45% β-sheet, 45% α-helical and 9% random coil).

The conformation of **P1**, **pL1**, **pL2** and **pL3** was compared with the predicted conformation structure of parent epitopes 1–3 derived from MSP-1 and RESA by I-TASSER online server (Table 2). The conformation of vaccine components (**P1**, **pL1- pL3**) did not match with the parent epitopes 1–3. Therefore, we have also investigated the conservation of epitopes between parasite species. MSP-1 and RESA protein sequences from both *P*.*yoelii* and *P*. *falciparum* species were aligned using ClustalW and resulted in overall sequence similarity score of 25% and 16% (S7 and S8 Figs).

**Table 2:** The secondary structures of native epitopes and their polyleucine conjugates.

Peptides	α helix	β sheet	Random coil
**P1**	22%	0%	78%
Epitope-1	100	0%	0%
**pL1**	72%	0%	28%
Epitope 2	91%	0%	9%
**pL2**	26%	72%	2%
Epitope 3	55%	0%	45%
**pL3**	9%	46%	45%

### Antibody responses

**Fig 4:**
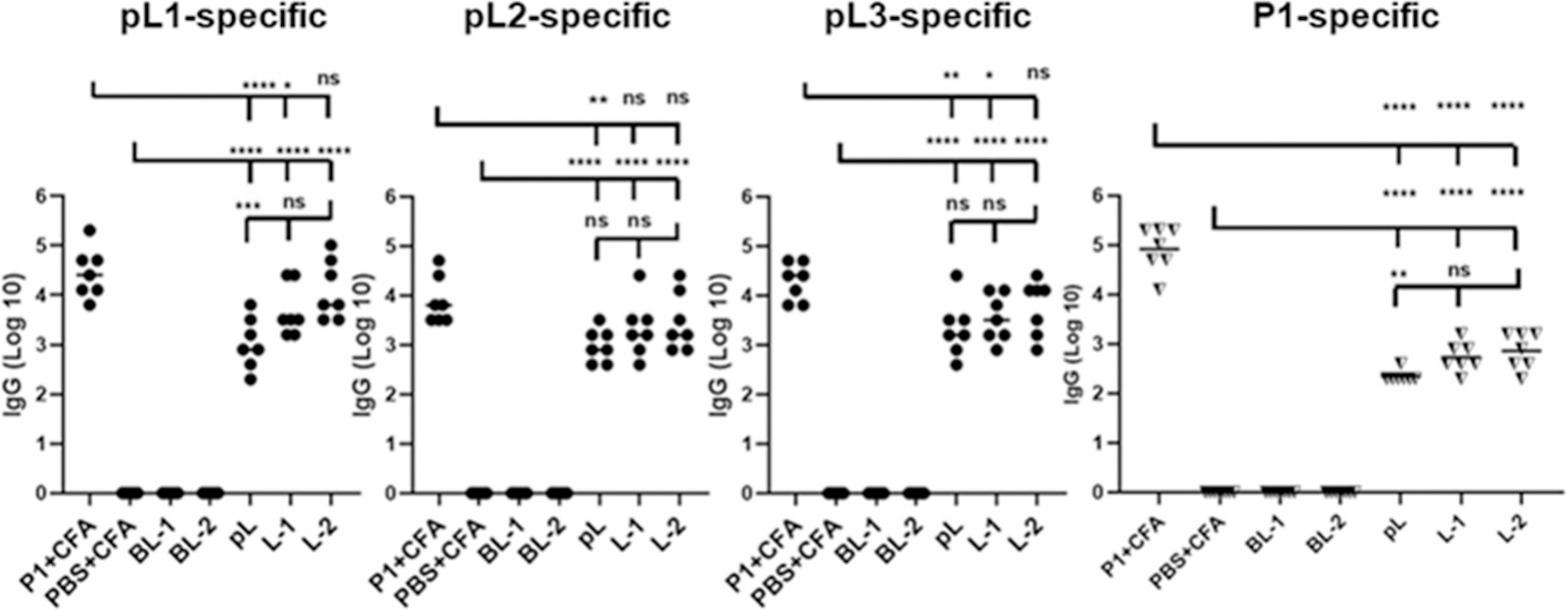
Production of antibody titres against polyleucine-conjugated (pL1-pL3) and P1-specific peptides. The mean value of each group is represented by a horizontal bar. The mean titre of each new vaccine group was compared to that of the positive (**P1+CFA**) and negative control (**PBS+CFA**) groups. Statistical analysis was performed by one-way ANOVA followed by Dunnett’s multiple comparison test; *, p < 0.05; **, p < 0.01; ***, p < 0.001; ****, p < 0.0001; ns: Not significant (p > 0.05). (n = 7 mice/group).

Female BALB/c mice were selected for the immunization study for congruency with the previous P1 study [10]. All mice received a single primary immunization (on day 0) and two boosts (on days 21 and 42) via the subcutaneous route. In this study, the previously reported vaccine candidate (**P1+CFA**) was used as a positive control, while **PBS+CFA**, **BL-1** and **BL-2** were used as negative controls. Peptides **pL**, **L-1** and **L-2** were administered as new vaccine candidates. The production of **P1**, **pL1**, **pL2**, and **pL3**-specific antibodies (IgG) was measured using an ELISA (Fig 4). Following the second boost, sera from **pL**, **L-1** and **L-2** immunized mice showed significantly higher **pL1**, **pL2** and **pL3**-specific antibody titres compared to the negative control (PBS+CFA). While **pL** and **L-1** induced significantly lower antibody titres compared to the positive control (**P1+CFA**), **L-2**-immunized mice produced similar epitope-specific IgG titres to **P1/CFA**. All new vaccine candidates (**pL1-pL3**) produced lower **P1**-specific IgG titres than **P1+CFA** immunized mice.

### Malaria challenge experiment

**Fig 5:**
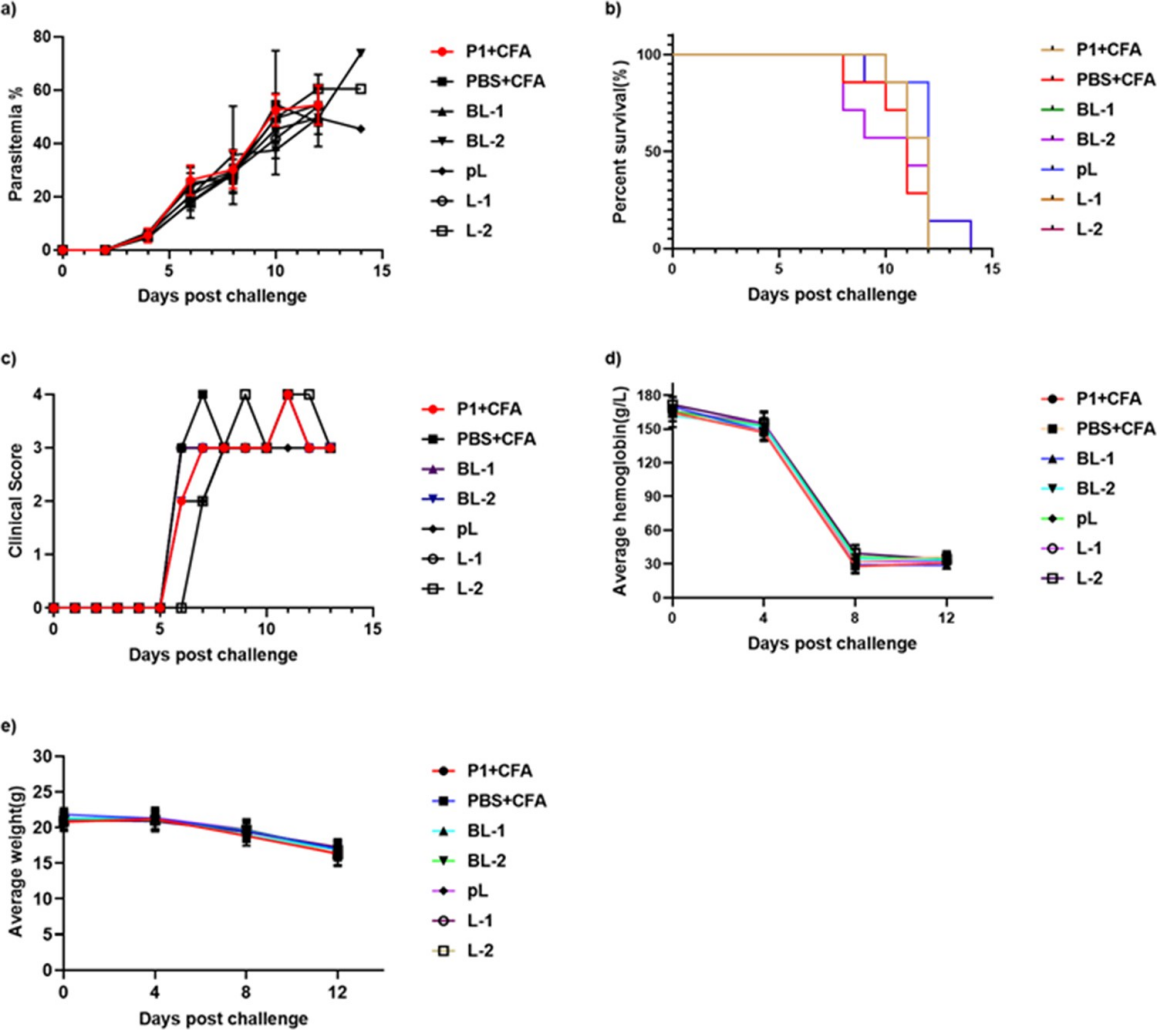
The new *Plasmodium falciparum* vaccine constructs failed to provide protection to mice following *Plasmodium yoelii* 17X (PY17X-NL) challenge. a) parasitemia curve; b) survival percentage; c) clinical score; d) average haemoglobin; and e) average weight following injection of *Plasmodium yoelii*. No statistically significant differences were observed between the groups. N = 7 mice/group. Data is presented as mean ± SEM.

The parasitemia, survival, clinical scores, haemoglobin level and body weight of all the immunized mice were monitored post-challenge until all mice were euthanised (Fig 5). The mice developed progressively higher parasitemias post-challenge until all immunized mice were euthanised by day 14 (Fig 5A and 5B). All immunized mice showed peak clinical scores of 3 to 4 (Fig 5C). At the time of challenge, the average hemoglobin levels were between 150–180 g/L; these gradually deceased following challenge in all groups to 30–45 g/L by day 12 (Fig 5D). Weight loss was also observed in all mice following challenge (Fig 5E).

## Discussion

A multiple antigen peptide, **P1** composed of epitopes derived from *P*. *falciparum* MSP-1 and RESA (Fig 1A) produced high IgG titres against epitopes in mice when administered with CFA, as reported previously [9,10]. When the vaccine efficacy was investigated against *P*. *yoelii*, **P1/CFA** showed promising protective antimalarial efficacy [10]. However, this vaccine construct cannot be used for further preclinical vaccine development as CFA is toxic [34,35]. To overcome this problem, we designed three CFA-free vaccine candidates **pL**, **L-1**, and **L-2** (Fig 2). The vaccine construct, **pL** contained a mixture of polyleucine-conjugated epitopes **pL1-3**, **L-1** anchored in mannosylated liposomes carrying **ML-1** and **L-2** anchored in mannosylated liposomes carrying **ML-2**. The vaccine candidate **L-2** was very effective in inducing IgG production and the produced titres against all three epitopes were equivalent to those induced by CFA-adjuvanted **P1** (Fig 4). Therefore, mannosylated liposomes anchoring polyleucine-conjugated antigens were more effective than polyleucine conjugates antigens alone. However, **P1**-specific IgG titres induced by the new vaccine candidates, including **L-2**, were significantly lower than those induced by **P1+CFA**, suggesting that a large portion of **pL**, **L-1** and **L-2** induced antibodies do not recognise multiepitope P1.

Moreover, despite the production of high antibody titres, **pL**, **L-1** and **L-2** did not protect immunized mice against parasite challenge (Fig 5). More surprisingly, even the previously reported vaccine candidate, **P1** failed to provide protection to immunized mice. The antibodies generated against epitopes 1–3 and even against **P1** were completely ineffective in providing any protection against challenge with the *P*. *yoelii* 17X parasite. One possible explanation for this unexpected outcome is the conformational constraints of B cell epitopes. B cell epitopes must maintain the conformational properties of the native protein to generate effective humoral immune responses [36,37]. However, the three-dimension (3D) Xray structures of MSP-1 and RESA are unknown; therefore, we used the I-TESSER online server to predict the whole 3D structure of these proteins along with the polyleucine-conjugated epitopes derived from MSP-1 and RESA.

According to the models, epitopes 1–3 derived from MSP-1 and RESA (Fig 1A) were mostly α-helical within the native proteins (Table 2). The conformation of polyleucine-conjugated epitopes (**pL1-pL3**) did not fully match the conformational structure of the parent epitopes (Table 2). Generally, polyleucine enhances the helicity of conjugated epitopes [38]. However, here, conjugation with polyleucine failed to induce helicity in the peptide epitopes (Table 2). This may explain why vaccine candidates that triggered the production of high levels of epitope-specific antibodies failed to protect mice against parasite challenge. There is a possibility, the 3D computational prediction of MSP-1 and RESA structures may have been inaccurate or an amino acid other than leucine should be used to control epitope conformation. Longer peptide epitopes (> 20 amino acids) [14,28], epitopes flanked with α-helicity-inducing sequences [15] and epitope dimers [39] have previously been reported as effective solutions for the conformational demands of B-cell epitopes. However, in our study, even **P1** adjuvanted with CFA failed to provide protection against *P*. *yoelii* 17X challenge in mice [10]. Thus, we investigated the conservation of the epitopes between *P*. *falciparum* epitopes and *P*. *yoelii* 17X. Surprisingly, MSP-1 and RESA alignment showed that epitopes are not conserved between the two malaria species (S7 and S8 Figs). This explains why anti-P1 antibodies did not recognize *P*. *yoelii* antigens, and did not provide protection, contrary to the previously reported data [10]. Thus, our vaccine candidates could be only examined in challenge with *P*. *falciparum*, which is incompatible with current mice model.

## Conclusion

In summary, were developed several polyleucine-conjugated epitopes anchored into mannosylated liposomes as *P*. *falciparum* vaccine candidates against malaria. The vaccine candidates were efficient in triggering epitope-specific antibody production. Unfortunately, immunized mice were not protected against challenge with the rodent malaria parasite *P*. *yoelii* 17X. Surprisingly, the previously reported vaccine candidate (**P1+CFA**) also failed to deliver any protection in mice against *P*. *yoelii* infection. Potential serious flaws in the vaccine design based on MSP-1 and RESA were identified including conformation and sequence mismatch between proteins of different origin, and inadequacy of *Plasmodium yoelii*-based challenge for *P*. *falciparum* proteins derived vaccine. Thus, the developed here vaccine candidates could be only examined in challenge with *P*. *falciparum*.

## Statistical analysis

GraphPad Prism 8 software was used to analyse the data. A Kaplan-Meier curve was used to analyse the survival percentage of mice. Multiple comparisons were performed by Dunnett’s comparison test using one-way ANOVA. Data were considered significantly different at *p < 0.05, **p < 0.01, and ***p < 0.000; differences were non-significant at p > 0.05.

## Supporting information

S1 Fig ESI-MS and HPLC of pL1. (TIF)

S2 Fig ESI-MS and HPLC of pL2. (TIF)

S3 Fig ESI-MS and HPLC of pL3. (TIF)

S4 Fig ESI-MS and HPLC of P1. (TIF)

S5 Fig MALDI-TOF of ML-2. (TIF)

S6 Fig Particle’s size (nm) of a) BL-1, b) BL-2, c) L-1, d) L-2, and e) pL. (TIF)

S7 Fig Protein sequences alignment of *P*. *falciparum* and *P*. *yoelii* ring associated surface antigen (RESA) proteins using ClustalW server v2.1, yellow highlighted sequence showing lack of similarity of BT2 epitope (within P. falciparum RESA protein) to the corresponding sequence in *P*. *yoelii*. and *P*. *falciparum* RESA protein (UniProt P13830-1) sequence is only 14.7% similar to that of *P*. *yoelii* RESA protein (UniProt Q7RH32-1). (TIF)

S8 Fig Protein sequences alignment of *P*. *falciparum* and *P*. *yoelii* MSP-1 proteins using ClustalW server v2.1, yellow highlighted sequences showing lack of similarity of BT1 and B1 epitopes (within *P*. *falciparum* MSP-1 protein) to the corresponding sequence in *P*. *yoelii*. and *P*. *falciparum* MSP-1 protein (UniProt A0A024V850-1) sequence is only 24.8% similar to that of *P*. *yoelii* MSP-1 protein (UniProt P13828-1). (TIF)
