# Supplementary figures and images for: Investigation of liposomal self-adjuvanting peptide epitopes derived from conserved blood-stage Plasmodium antigens
Source: PLoS One. 2022 Mar 11;17(3):e0264961. doi: 10.1371/journal.pone.0264961 (PMC8916655; doi:10.1371/journal.pone.0264961)

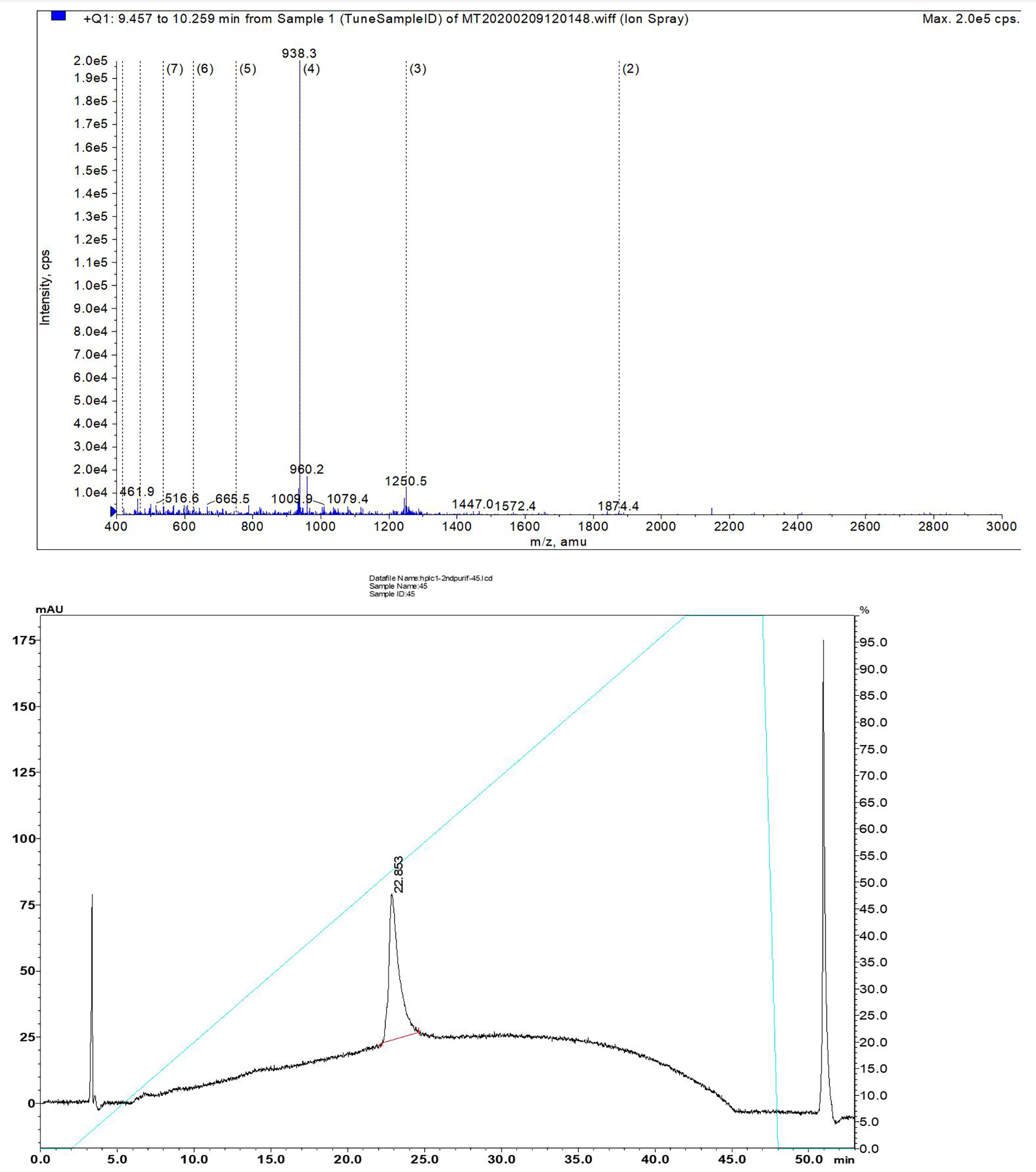

Supplement: S1 Fig — (TIF) [file pone.0264961.s001.tif]

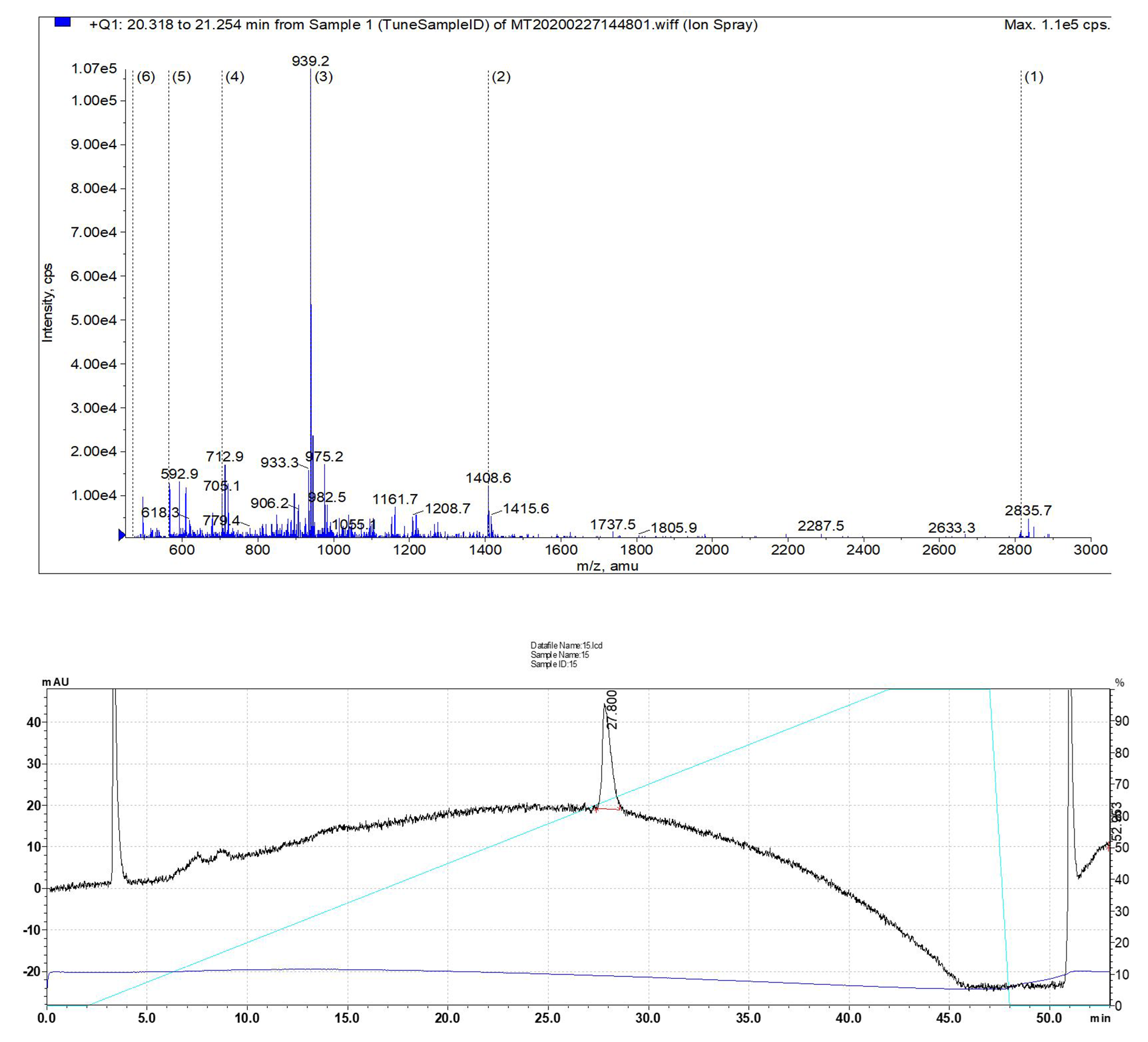

Supplement: S2 Fig — (TIF) [file pone.0264961.s002.tif]

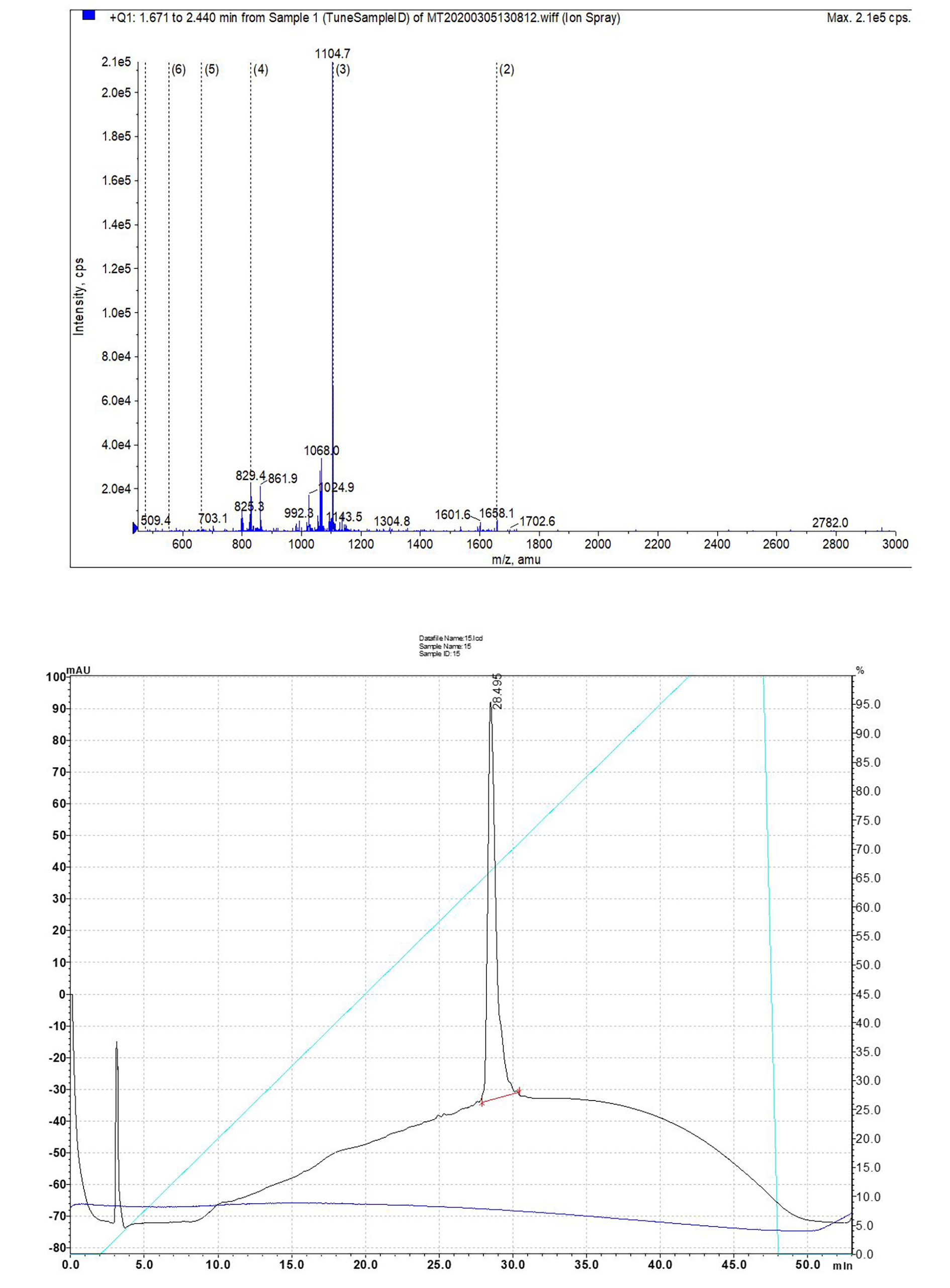

Supplement: S3 Fig — (TIF) [file pone.0264961.s003.tif]

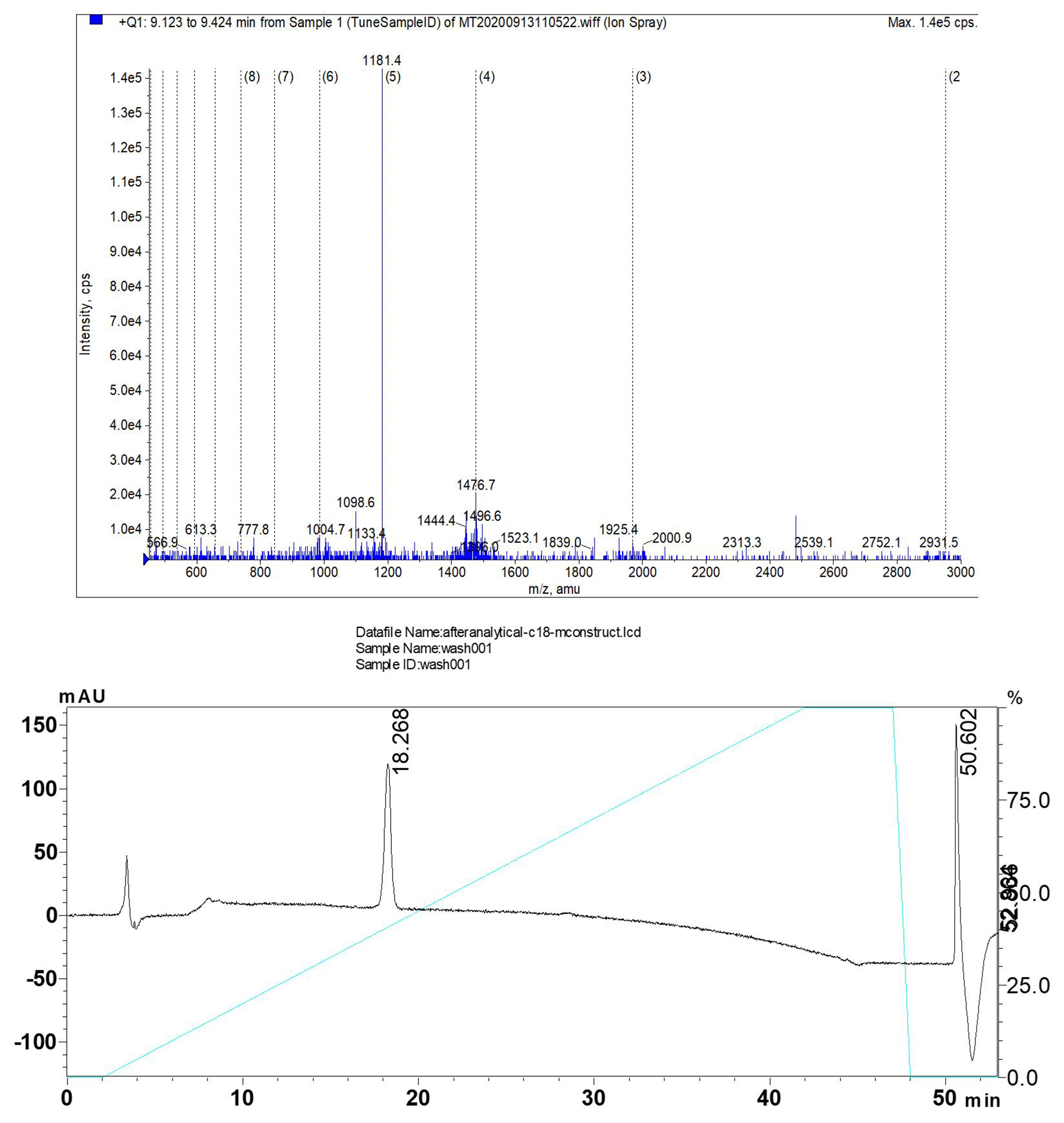

Supplement: S4 Fig — (TIF) [file pone.0264961.s004.tif]

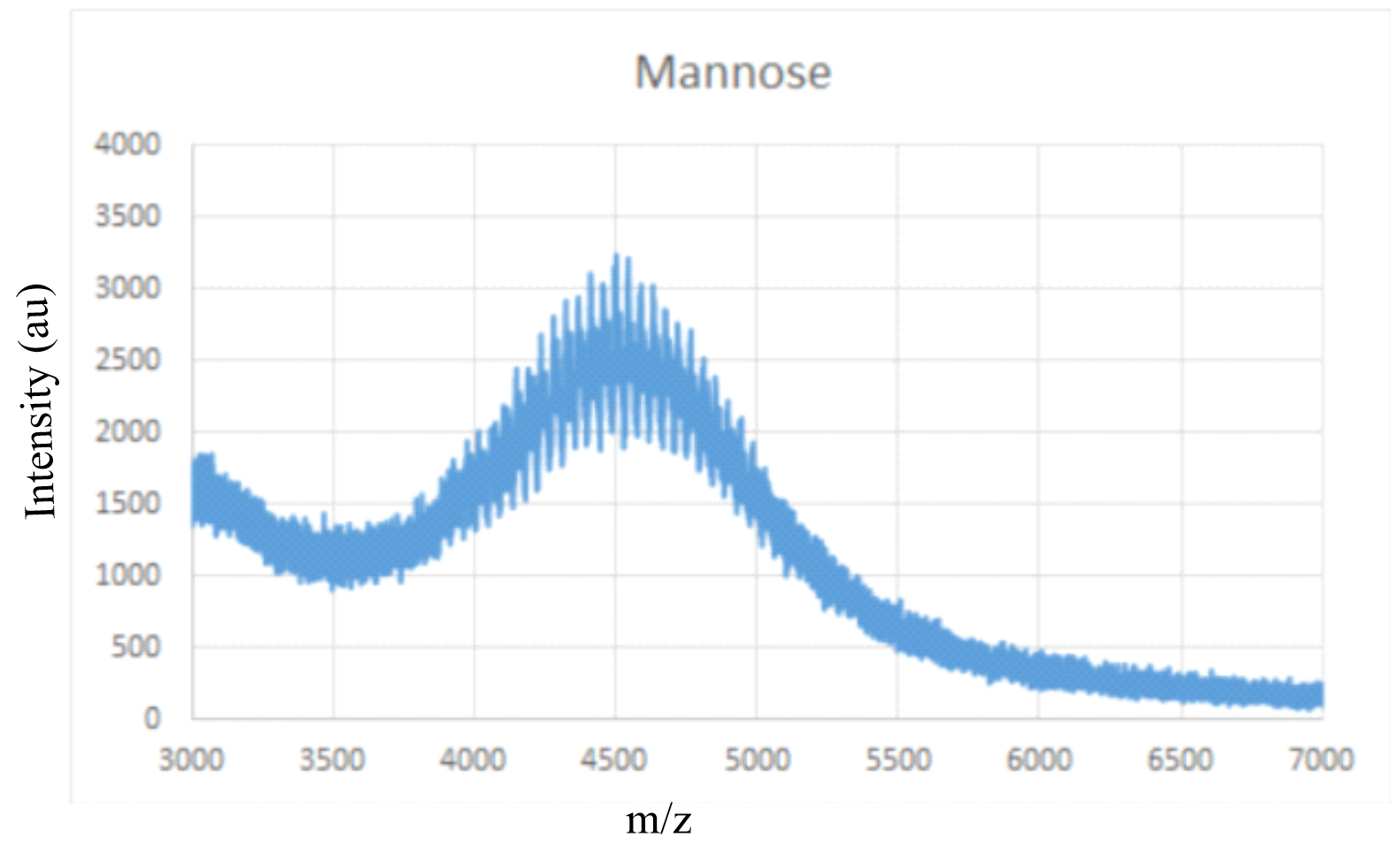

Supplement: S5 Fig — (TIF) [file pone.0264961.s005.tif]

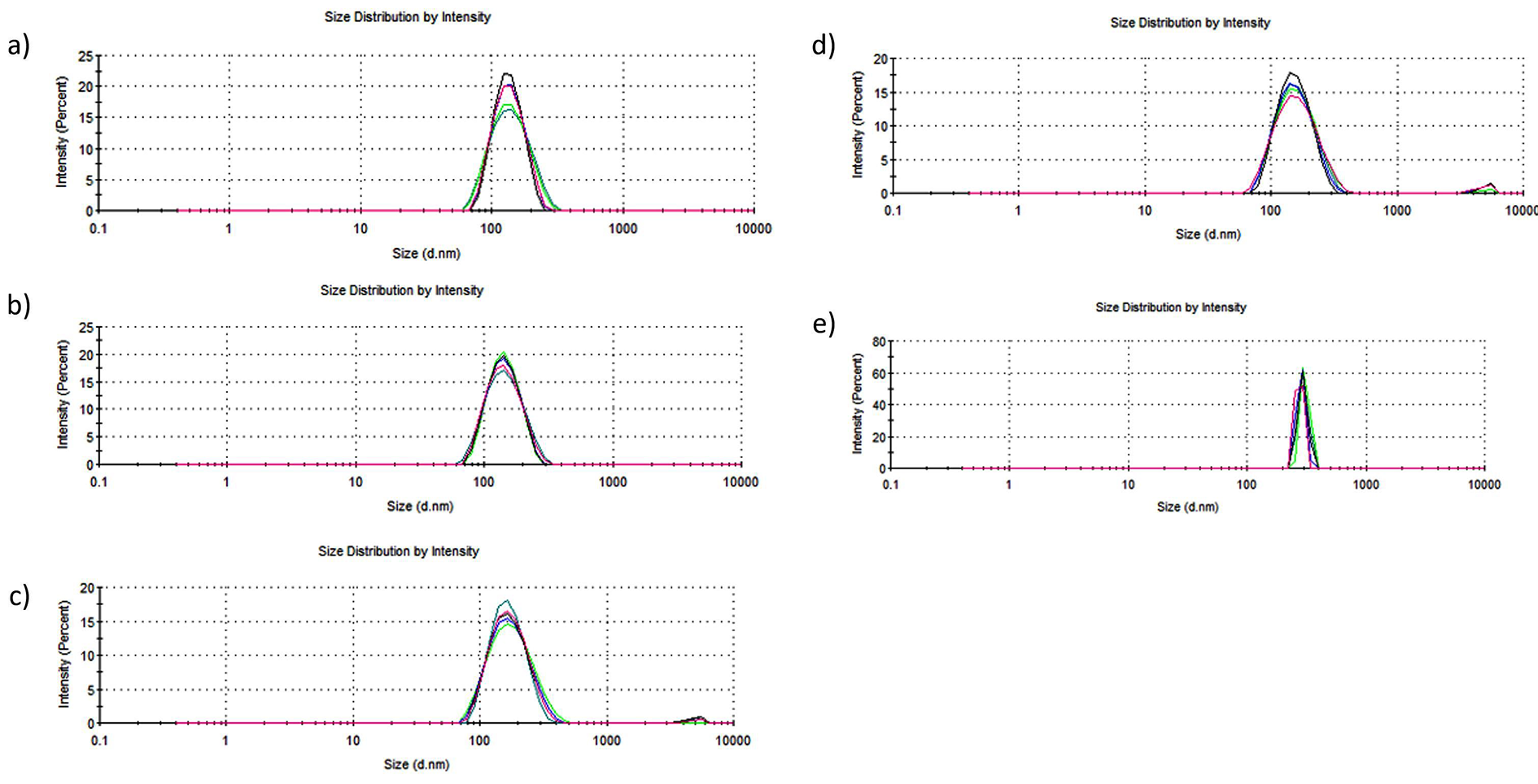

Supplement: S6 Fig — (TIF) [file pone.0264961.s006.tif]

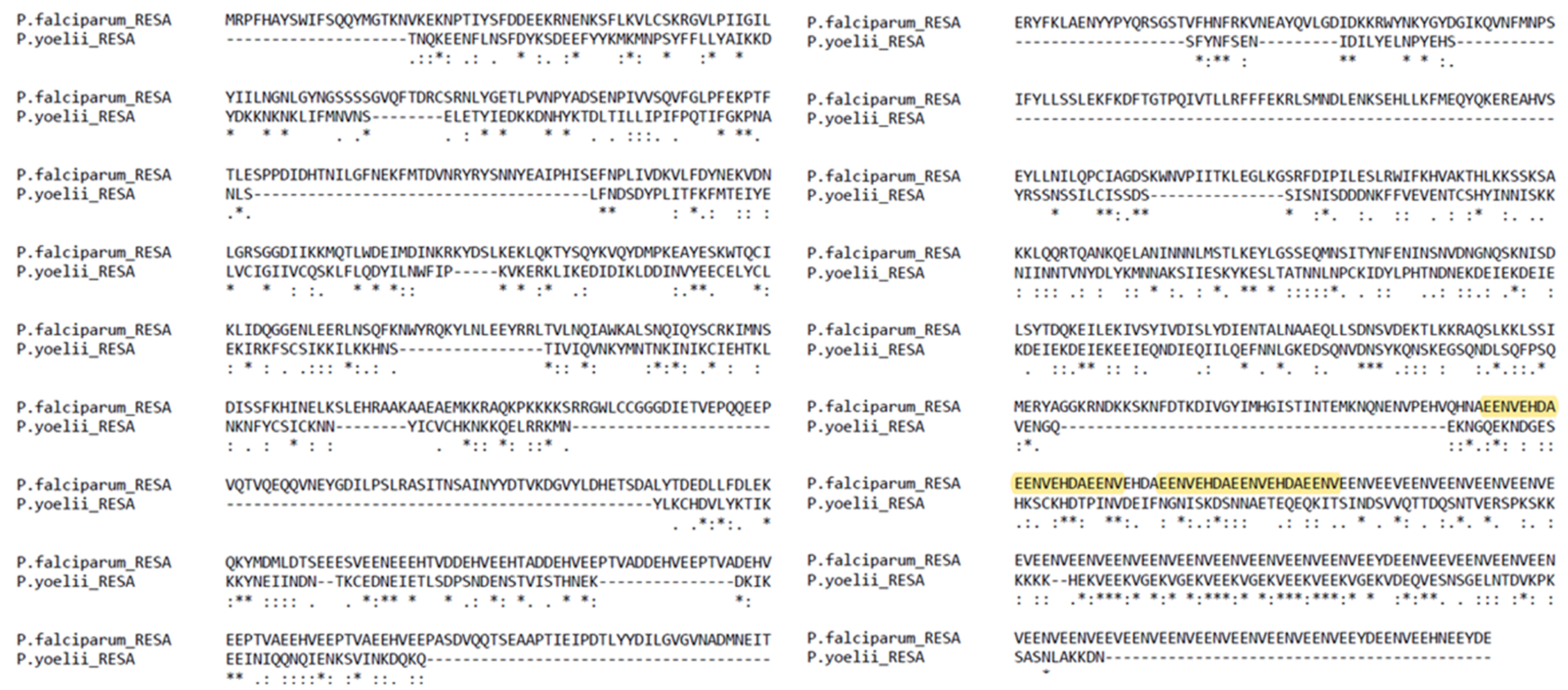

Supplement: S7 Fig — (TIF) [file pone.0264961.s007.tif]

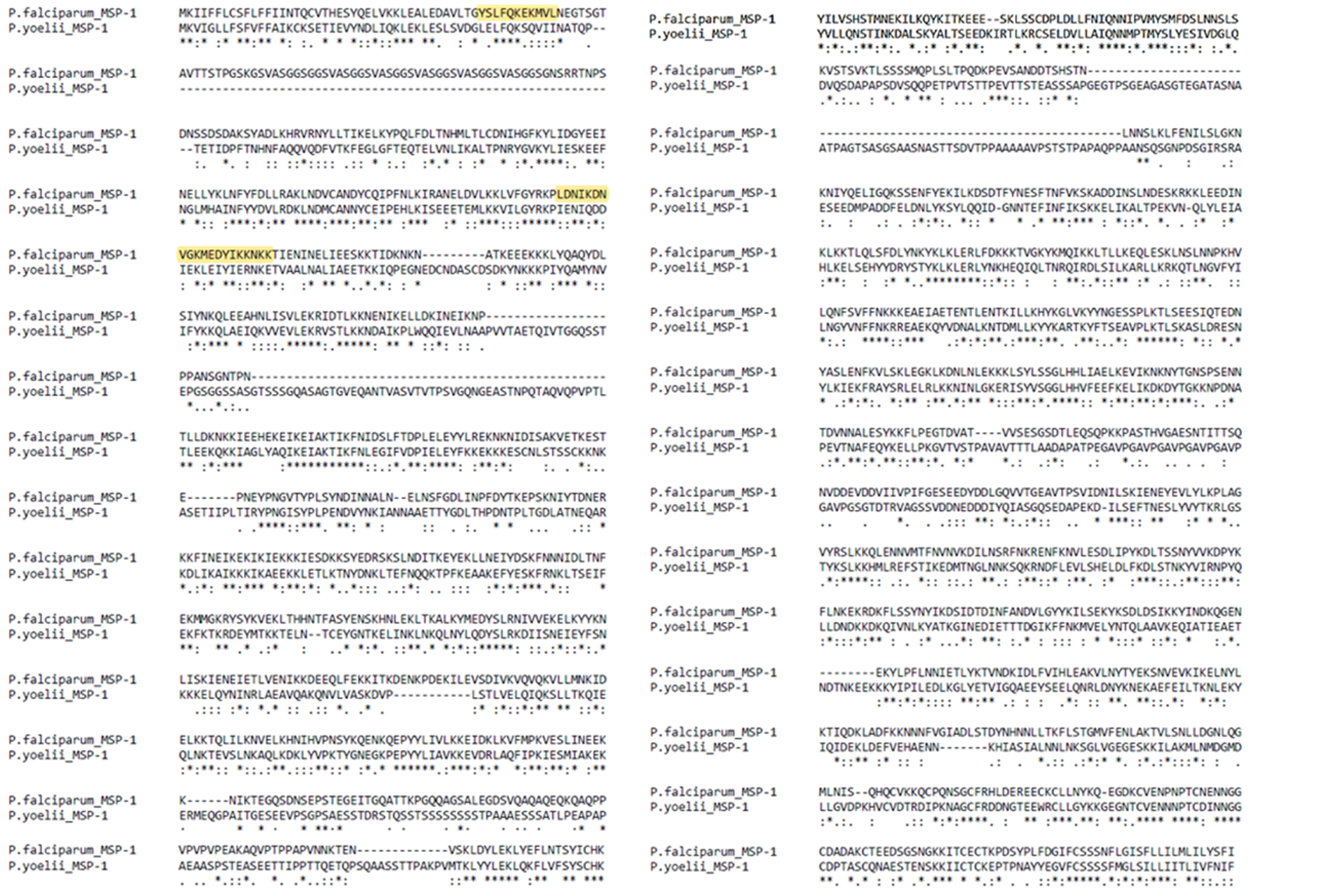

Supplement: S8 Fig — (TIF) [file pone.0264961.s008.tif]
